# The Role of T-Type Calcium Channel Genes in Absence Seizures

**DOI:** 10.3389/fneur.2014.00045

**Published:** 2014-05-09

**Authors:** Yucai Chen, William Davis Parker, Keling Wang

**Affiliations:** ^1^University of Illinois at Chicago, Peoria, IL, USA; ^2^Hebei Children Hospital, Shijiazhuang, China

**Keywords:** absence epilepsy, T-type Ca^2+^ channels, genetics, expression, methylation

## Abstract

The thalamic relay neurons, reticular thalamic nucleus, and neocortical pyramidal cells form a circuit that sustains oscillatory burst firing, and is regarded as the underlying mechanism of absence seizures. T-type calcium channels play a key role in this circuit. Here, we review the role of T-type calcium channel genes in the development of absence seizures, and emphasize gain or loss of function mutations, and other variations that alter both quantity and quality of transcripts, and methylation status of isoforms of T-type calcium channel proteins might be of equal importance in understanding the pathological mechanism of absence seizures.

## Introduction

Genetic generalized epilepsies (GGE) are regarded as primarily GGE, which are frequently expressed sporadically in a population. Many genetic generalized epilepsies feature absence seizures (GGE-AS) including childhood absence epilepsy (CAE), juvenile absence epilepsy (JAE), juvenile myoclonic epilepsy (JME), and others. Absence seizures are brief, generalized epileptic seizures of sudden onset and termination. Impairment of consciousness and generalized spike-and-slow wave discharges (SWD) on EEG are two essential feature of absence seizure (1).

The neurons in the reticular thalamic nucleus (RT), thalamic relay neurons [thalamocortical (TC) neurons], and neocortical pyramidal cells make up a circuit that takes part in the formation of sleep (TC circuits). Studies have shown that TC circuits control the rhythm of cortical excitation by the thalamus, and underlie normal physiologic activities such as those occurring during sleep. A change in this circuitry is thought to be involved in the mechanism of SWD, and absence seizures (2–4).

Hypothalamic relay neurons can activate cortical pyramidal neurons both in the tonic mode, and burst model: tonic mode occurs at awakening and during rapid eye movement (REM) sleep. Burst mode occurs during non-REM sleep (5). The burst mode is generated by T-type calcium channels, which allow for low-threshold depolarization of neurons, creating bursts of action potentials through voltage-gated sodium channels (6).

Many data suggest that abnormalities in T-type calcium channels may be the primary pathogenic event in absence seizures. In the brain, different T-type calcium channel genes are co-expressed in a single neuron, e.g., *Cav3.2* and *Cav3.3* are co-expressed in RT neurons, where they are thought to be responsible for generating the SWD seen in absence epilepsy. The same subset of T-type calcium channels may have different alternative isoforms, which appear to be unique to each individual. These unique isoforms might have specific biophysical properties such as voltage-dependency of channel activation, inactivation, deactivation, and recovery from inactivation.

It is extremely complicated when variable amounts of different T-type calcium channel splice variants are co-expressed in one neuron because very subtle modifications in T-type calcium channels can drastically affect the physiological responses of thalamic neurons, and play a pathogenic role in the initiation of absence seizures (7). Here, we review the role of T-type calcium channel genes in the pathogenesis of absence seizures, and emphasize that variants of these genes may initiate seizures through effects on the function or amount of the resulting protein either through modification of the primary protein structure or through modifications brought about by altered transcription.

## Complex Genetics of GGE with Absence Seizures

Evidence strongly supporting a role for genetic factors in many types of GGE-AS comes from twin studies with reported concordance rates consistently higher in monozygotic than in dizygotic twins (8, 9). CAE represents one of the typical examples of these genetically determined GGE. There is a 16–45% positive family history in CAE patients. However, the penetrance of CAE is incomplete, with the concordances of monozygotic twins being 70–85%, and first-degree relatives being 33% (10). GGE-AS is complex genetic epilepsy, which is expressed sporadically and whose phenotype does not follow simple Mendelian inheritance.

The genetics involved in absence seizure are very complicated and many factors may work together to contribute to the phenotype of GGE-AS (11). Direct sequencing of candidate genes combined with a functional study of the abnormal genes products *in vitro* has met some success in identifying GGE-AS’s susceptibility genes (12). These factors include mutations of the *Cav3.2* and *GABRB3* gene (the gene encoding the gamma-aminobutyric acid receptor subunit beta-3 protein) in patients with CAE (13, 14) and mutations of *SLC2A1* (the gene encoding the glucose transporter protein type 1) in early-onset absence epilepsy (under 4 years of age) (15, 16). Moreover, copy number variants have been found to play an important role in the patients with epilepsies (17, 18). However, these changes discovered thus far occur in only a small portion of patients with epilepsy. Sequence studies in quite numbers of ion channel genes in individuals with sporadic epilepsy and in unaffected controls found that both groups had similar numbers of rare missense variants in these epilepsy-associated ion channel genes, demonstrating that single variants in epilepsy genes might have poor predictive value in the formation of epilepsy (19). Moreover, an experiment using exome sequencing followed by large-scale genotyping cannot reveal that single rare variants of genes have large effect in idiopathic generalized epilepsy (20). These results suggest that rare variant might be not the major factor in the mechanism of epilepsy, it also reflects a mistake concepts of pathogenic mechanisms of epilepsy, in which, we may have placed excessive emphasis on the role of single missense mutations and neglect the effects of altered transcription levels or alternative transcription in the pathogenesis of GGE-AS.

In exploring the genetic basis of GGE with absence seizures, it is important to realize that typical pure absence seizures and atypical absence seizures may have different origins. Typical pure absence seizures are characterized by: (1) onset in early life, and may remit in the adult. (2) Brief absence seizures that occur frequently, sometimes hundreds per day, characterized by 3 Hz spike wave on EEG. (3) No radiological findings or neurological abnormalities. (4) The symptoms can be activated by hyper-ventilation or light stimulation (21, 22). These unique characteristics appear in typical absence seizures and suggest that there are genes, which play a role in the maintenance of proper TC synchronization and that expression may be age-specific. The expression of these genes or biological characteristics of their expressed proteins may be temporarily activated by environmental factors such as hyper-ventilation or light stimulation. On the other hand, patients with atypical absence seizure, usually have other types of epilepsies such as atonic, tonic, and myoclonic. These patients usually have abnormal cognitive and neurologic function. In these patients, the EEG typically appears slow (<2.5 Hz) SWD, with polymorphic, often asymmetrical discharges that may also include irregular spike and slow wave complexes, and fast and other paroxysmal activities. Atypical absence seizures cannot be easily provoked by hyper-ventilation or light stimulation (23). The origin of atypical seizures may result from structural abnormalities in the brain or from complications from other non-neurologic disorders. Genetic factors involved in atypical absence seizure may have far reaching impact on brain development leading to the other forms of neurologic dysfunction and may have lifelong expression. An example of this is the mutation of *CACNA1A*, which has a persistent impact on brain development and is involved in a spectrum of different clinical phenotypes, including episodic ataxia type 2, autosomal dominant spinocerebellar ataxia, hemiplegic migraine-1, and absence seizure (24).

## Current Knowledge of Mechanism of Absence Seizures

**Figure 1:**
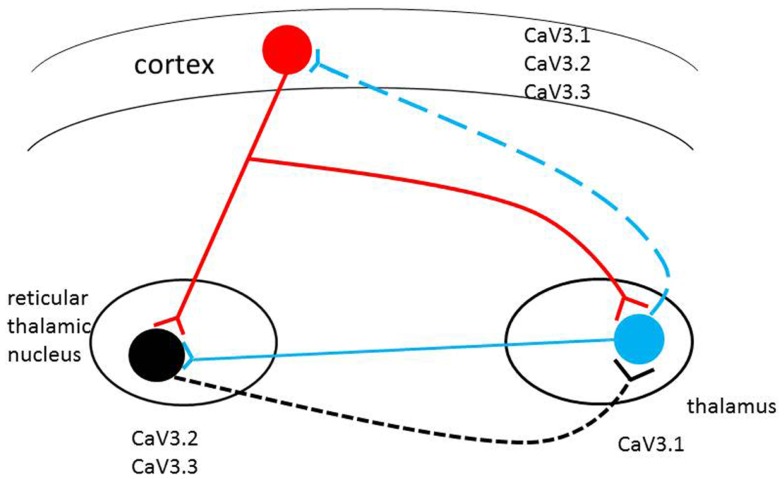
**Schematic representation of the thalamocortical circuit and T-type Ca^2+^ channel expression**. Cav3.2 and Cav3.3 channels are mainly expressed in RT (reticulothalamic) neurons; Cav3.1 channels are highly expressed in thalamocortical (TC) neurons. All three T-type calcium channels are found in different but overlapping in layers of the neocortex. Alterations in level of expression or biophysical properties of the T-type calcium channel genes (single/multiple missense mutations, alternative transcripts, epigenetic factors) in the circuit may induce pathophysiological changes in the brain with the potential to induce absence seizures.

Cortical neurons directly innervate reticular (RT) and TC neurons. The RT neurons supply GABAergic projections onto each other and onto TC neurons, which in turn TC neurons onto neocortical neurons. Both neocortical and TC cells have excitatory projections back onto RT neurons, and the activity of the circuit is further revised via thalamic local circuits and neocortical interneurons (Figure 1) (2, 25). The circuit of RT, TC, and neocortical pyramidal cells make up a circuit, is implicated in the formation of sleep spindles and of SWD, the mechanism of absence epilepsy (26). Thalamic neurons fire in two different modes, burst or tonic (27, 28). The state of the neurons decide the mode of firing: hyperpolarization by inhibition leads to burst firing, and depolarization by excitation leads to tonic firing (28). T-type calcium channels are known to play a key role in generation of neuronal burst firing (29). Influx of calcium ions through T-type calcium channels results in depolarization of the membrane, allowing T-currents to generate low-threshold spikes, which then touch off the bursts of sodium-dependent action potentials. The T-type calcium channels must first be released from inactivation by membrane hyperpolarization to less than the normal resting potential. They can then be activated by a tiny degree of depolarization driven by hyperpolarization-activated current. When activated, T-type channels produce low-threshold calcium currents, which incur a rebound plateau excitation above the sodium action potential threshold, and result in the generation of a burst of action potentials (30, 31). RT neurons are regarded as pacemakers of spindles. The concept is based on the absence of oscillations in TC systems after disconnection from the RT nucleus and the presence of spindle rhythmicity in the deafferented RT nucleus (2, 32). Moreover, data have shown that an alteration in the firing properties of TC nuclei, caused by a disruption of the Cav3.1gene in mice, is sufficient to induce absence seizures (33–35). More recent results showed that TC oscillations could be initiated by cortical inputs via the cortico-TC–nRT–TC pathway in the Gria4^–/–^ model of absence epilepsy (36).

## T-Type Calcium Channel Genes and Their Expression

Three different T-type calcium channels express T-currents: alpha 1G (*CACNA1G*, Cav3.1), alpha 1H (*CACNA1H*, Cav3.*2*), and alpha 1I (*CACNA1I*, Cav3.3) (6, 37–39). Of these three T-type calcium channels, alpha1G subunits are the main source of T-type channels in TC relay nuclei; alpha1H and alpha 1I subunits are co-expressed in thalamic reticular nucleus (RT), and all three types are found in different but overlapping layers of the neocortex (40). All three T-type calcium channel genes have multiple transcripts, and these isoforms likely have different biophysical properties, accounting for the complex genetic characteristics of absence seizure. The differences in the electrophysiological properties of the isoforms of T-type channels suggest that the firing patterns of neurons may depend on the expression levels of different isoforms of T-type channels. It is important to understand the expression profiles of the isoforms of three sub-types in particular neurons, and to examine differences in their electrophysiological properties in order to understand their associated neuronal activities (41). Both Cav3.2 and Cav3.3 are co-expressed in the RT neurons. It has been established that Cav3.3-mediated currents generate bursts that are most closely correlated with typical RT bursts (42). However, Cav3.2 channels are also expressed in the RT and Cav3.2-like currents are observed in these neurons. The fast activation kinetics and the lower threshold of activation in Cav3.2 channels may suggest that this channel plays a critical role in the initiation of bursts (42).

Subtle modifications in T-type channel gating have significant consequences in the TC circuits (7). Individuals may have diverse T-type calcium isoforms; if some of these unique isoforms change the physiological characteristics of channels, they may facilitate aberrant cortical synchronization and possibly initiate absence seizure. Therefore, the inherited complexity of multiple variants of the T-type calcium genes may be expressed through different T-type calcium channel gene patterns to initiate CAE development (see Figure 1).

## Animal Experiments Indicate T-Type Calcium Channel Involvement in the Formation of Absence Seizures

### Absence seizure animal model GAERS has alternative alpha1 H transcripts and selectively increases T-current in RT neurons

Genetic absence epileptic rats from Strasbourg (GAERS) have recurrent generalized non-convulsive seizures characterized by bilateral and synchronous SWD, accompanied by behavioral arrest, staring, and sometimes twitching of the vibrissae. In GAERS, drugs effective against absence seizures in humans can also inhibit the SWD in a dose-dependent manner, but not the drugs specific for human’s convulsive or focal seizures (43). The many similarities between GAERS and human absence seizures support using this type of genetic model for the study of human absence seizures. Early research showed that the T-type calcium current is selectively increased in the RT neurons of GAERS (44). Quantitative *in situ* hybridization demonstrated small but significant elevations in T-type calcium channel mRNA (alpha1g and alpha1h) in the thalamus of GAERS (45). Moreover, a mutation, R1584P, located at Exon24, which encodes a portion of the III–IV linker region, of the Cav3.2 gene, has been found in the GAERS (46). In GAERS, there are two major Cav3.2 splice variants, characterized by inclusion or absence of Exon25. The presence of Exon25 is required to produce significantly fast recovery from channel inactivation and great charge transference during high-frequency bursts. Of particular interest, the ratio of Cav3.2 (with 25 Exon) mRNA to Cav3.2 (without 25 Exon) mRNA is greater in the thalamus of GAERS animals compared with non-epileptic controls, which may suggest that the R1584P mutation have a role in adjusting the relative proportion of splice variants, and work in a mechanism of absence seizure (46).

### Absence seizures associated with mutations of cacna1a, and cacna1a’s ancillary calcium channel subunits in mice are the results of indirect potentiation of T-type calcium current

The *CACNA1A* (Cav2.1), coding the alpha 1A subunit protein, which mediates the entry of calcium ions into excitable cells. Alpha 1A is involved in many calcium-dependent processes, including muscle contraction, hormone, or neurotransmitter release (47). Mutations in *Cav2.1* are related to episodic ataxia type 2, familial hemiplegic migraine type 1, and spinocerebellar ataxia type 6, and some of these individuals have absence epilepsy (48). Several spontaneously occurring homozygous mouse mutants of Cav2.1 are good research models of human absence epilepsy, including tottering, leaner, rocker, rolling nagoya, lethargic, ducky, and star-gazer (49–53). These strains exhibit episodes of motor arrest with spike–wave EEG similar to that seen in human absence epilepsy, but also show cerebellar degeneration, ataxia, and dystonia. There is a 45% increase in peak current densities of T-type calcium channels currents in tottering (*Cav2.1*/alpha 1A subunit), lethargic (β4 subunit), and star-gazer (γ4 subunit) mice compared with wild type, demonstrating that a mutation in Cav2.1 or in its regulatory subunit genes increases intrinsic membrane excitability in thalamic neurons by potentiating T-type calcium channel currents. Another example of cacan1a mutations that correlate with absence seizure comes from the Cav2.1 knockout (KO) mice. Mice with a null mutation of Cav2.1 are susceptible to absence seizures characterized by typical SWD and behavioral arrests. Isolated TC relay neurons from this type of KO mice show increased T-type calcium currents *in vitro* (35).

### Mice with genetically modified T-type calcium channels show aberrant electrophysiological properties implicated in the pathogenesis of seizures

#### Cav3.1 genetically modified mice

Mice with a null mutation of the Cav3.1 of the T-type calcium channel lack the burst mode firing action potential, but show the normal pattern of tonic mode firing. The thalamus in these Cav3.1-deficient mice is specifically resistant to the generation of SWD in response to GABA (B) receptor activation. Therefore, Cav3.1 T-type calcium channels are thought to play a critical role in the genesis of absence seizures in the TC circuit (34, 35). This idea was further supported by studies of Cav2.1 (−) and Cav3.1 (−) double mutation mice, which demonstrate that generation of SWD in these mice is suppressed by the exclusion of the cacna1g gene. Offspring of cross-bred Cav2.1^−/−^ mice with mice harboring a null mutation in Cav3.1 show a complete loss of T-type calcium current in TC neurons and display no SWD. Similar results were obtained using double-mutant mice characterized by a Cav3.1 mutation plus another mutation such as lethargic [beta4 (lh/lh)], tottering [alpha1A (tg/tg)], or star-gazer [gamma2 (stg/stg)] (35). The critical role of cacna1g in the absence seizure was further supported by an experiment showing that two BAC transgenic murine lines, which overexpress the cacna1g gene, induce pure absence epilepsy through genetic enhancement of the TC network (33).

#### Knockout of Cav3.2

In Cav3.2 KO mice, the burst of RT neurons is characterized by a lower spike frequency and less prominent acceleration–deceleration change. In addition, Liao and colleagues found that the long-lasting tonic episodes in RT neurons in these mice exhibit less regularity than similar events seen in wild type mice (54). Another example of role of the Cav3.2 gene in epileptogenesis originates from studies of the pilocarpine model of epilepsy. In this model, there is a transient and selective upregulation of Cav3.2 subunits at both mRNA and protein levels after pilocarpine-induced status epilepticus. The development of some of the neuro-pathological features of chronic epilepsy such as subfield-specific neuron loss in hippocampal formation and mossy fiber sprouting are almost completely absent in Cav3.2 KO mice (55).

#### Knockout of Cav3.3

Two T-type calcium channel genes are expressed in the nucleus reticularis thalami (RT); Cav3.2 and Cav3.3, with the Cav3.3 protein being more abundantly expressed. In the Cav3.3 KO mice, the absence of Cav3.3 channels prevented oscillatory bursting in the low-frequency (4–10 Hz) range in RT cells while leaving tonic discharges unchanged. In contrast, adjacent TC neurons expressing Cav3.1 channels retained low-threshold bursts (56).

## Anti-Absence Seizure Drugs and T-Type Calcium Channel

T-type calcium channels play an important role in the generation and maintenance of SWD in absence seizure making them potential therapeutic targets. Drugs inhibiting the function of these three subtypes of T-type calcium channels might have a role in the treatment of absence seizures through inhibition of oscillatory thalamocortical circuits. Ethosuximide (ETX), for example, a block of all three T-type Ca^2+^ channels, is considered as first choice drug for treating absence seizures (57). A new study identified two T-type calcium channel blockers, Z941 and Z944, with attenuated burst firing of thalamic RT neurons in GAERS. Z941 and Z944 potently suppressed absence seizures by 85–90% and reduced both the duration and the cycle frequency of the SWD in GAERS. It was suggested that Z941 and Z944 likely target the TC circuits involved in SWD by inhibiting the ictogenic properties of the cortical neurons, as well as by disrupting the resonant circuitry of the TC and RT neurons (58). The ability of the T-type calcium channel antagonists to inhibit absence seizures and reduce the duration and cycle frequency of spike-and-wave discharges, also suggests that T-current generated by T-type calcium channels is a key component in the formation of absence seizures.

## Variants Detected in T-Type Calcium Genes Involving in the Mechanism of Absence Seizure

### Variants of Cav3.2 gene detected in patients with CAE

Chen et al. conducted direct sequencing of exons 3–35 and the exon–intron boundaries of the Cav3.2 gene in 118 CAE patients of Han ethnicity recruited from Northern China. Sixty-eight variations were detected in the Cav3.2 gene. Of the variations identified, 12 were missense mutations and were found in a heterozygous state in 14 of the 118 patients, but in none of the 230 unrelated controls. The identified missense mutations occurred in highly conserved residues of the T-type calcium channel gene. Computer simulations predicted that some mutations would favor burst firings. These mutations were introduced into human Cav3.2 cDNA and transfected into HEK-293 cells for whole-cell patch-clamp recordings. These recordings showed that many mutant channels are activated in response to a smaller voltage change, a change in the rate of recovery of channels from their inactivated state, or an increase in the surface expression of the channels (59–62). More recently, experiments suggested that gain-of-function mutations in Cav3.2 T-type Ca^2+^ channels increase seizure susceptibility by directly altering neuronal electrical properties and indirectly by changing gene expression (63).

Research results from Heron et al. support the original concept that Cav3.2 gene is a susceptibility gene in absence seizure and is also associated with an extended spectrum of idiopathic generalized epilepsies in the Caucasian population. In their study, 240 epilepsy patients and 95 control subjects were tested. More than 100 variants were detected, including 19 novel variants involving amino acid changes in subjects with phenotypes including childhood absence, juvenile absence, juvenile myoclonic, and myoclonic astatic epilepsies, as well as febrile seizures and temporal lobe epilepsy. Electrophysiological analysis of 11 variants showed that 9 had altered channel properties, generally in ways that should increase calcium current (64–66).

### Variations in Cav3.1 and Cav3.3 in patients with GGE

To evaluate the Cav3.1 (alpha 1G) gene’s contribution to the pathological mechanism of common CAE, Chen et al. sequenced all of the exons in the Cav3.1 gene in 48 Chinese patients with CAE, and failed to find a link between alpha 1G mutations and human absence epilepsy (59). However, a recent report described several putative functional variants of the Cav3.1 gene in patients with GGE. The Ala570Val variant was found in one of 123 GGE patients and was not observed in 360 healthy controls. The Ala1089Ser substitution segregated into three JME affected members of a two-generation Japanese family and in one healthy control. In addition, an Asp980Asn substitution was found in two JME patients and three control individuals (67). To date, no report has shown a link between patients with idiopathic epilepsy and mutations of Cav3.3 (68).

### Alternative transcripts of T-type calcium genes and absence seizure

Cav3.2 is located on chromosome 16p13.3. It is expressed in the thalamic reticular nucleus. It is extensively alternatively spliced and generates a family of variant transcripts. An analysis of 115 GenBank accessions from 104 alpha 1H cDNA clones, illustrates that the human Cav3.2 gene contains 38 introns with 10 different mRNAs, 7 alternatively spliced variants, and 3 unspliced forms. Zhong also found that the Cav3.2 gene is alternatively spliced at 12–14 sites, and is capable of generating both functional and non-functional transcripts (69). Biophysical analysis of different alternative Cav3.2 reveals variations in kinetics and steady-state gating parameters, which may result in an altered membrane firing. Zhong et al. further examined mutations of Cav3.2 gene, such as C456S, D1463N, and A1765A, which appear to be unique in Chinese CAE patients. These mutations, elicit minimal or no changes in Cav3.2 function resulting from candidate exonic splicing enhancer sequences. They demonstrated that these missense and silent mutations may create or change the regulatory specificity of predicted exonic splicing enhancer sequences that control splicing regulation (69). The Cav3.2 gene is highly variable. According to our data (59), and analysis of NCBI SNP data, the average SNP density of exons in the Cav3.2 gene is almost 1 SNP for every 48 base pairs. The average SNP density of introns in the Cav3.2 gene is almost one SNP for every 64 base pairs. It is 40 times higher in exons and 20 times higher in introns than the average genomic density. Many of these SNPs’ allele frequency rates are above 10%. A low/high frequency SNP rate comprises a complicated profile, where each individual may have unique SNP variants. The electrophysiological properties of alpha 1H subunit with a single mutation of G773D are different from those seen with combined mutations of G773D and R788C (62). In addition, an interaction between domains II and III of alpha1 H subunit may play a role in activation of the T-type Cav3.2 channel (70). Extensive variants of alpha 1H may suggest that there are different Cav3.2-like *T*-currents in each individual. In addition, variants of the Cav3.2 gene may destroy, create, or alter the regulatory specificity of predicted exonic splicing enhancer sequences that control splicing regulation. An example of this is found in the GAERS animal model where a point mutation (R1584P) in cacna1h gene can lead to formation of differentiate spliced mRNA. The presence of Exon25 in the transcript results in the production of faster recovery from channel inactivation and enhanced charge transference during high-frequency bursts (46). More research is required to clarify how variants of Cav3.2 gene affect their physiological characteristics and level of transcripts, and how these changes relate to the expression of absence seizures.

The two other sub-types of T-type calcium genes also have many alternative transcripts according to GenBank data: Cav3.3 gene produces 7 different mRNAs, and Cav3.1 gene produces 34 alternatively spliced mRNAs. These unique isoforms might have specific biophysical properties such as voltage-dependencies of channel activation, inactivation, deactivation, and recovery from inactivation (71). A very subtle change in the properties of the T-calcium current can drastically affect the physiological output of the thalamic neurons (7). It is therefore reasonable to speculate that some individuals have unique “strong” T-type calcium currents because of their distinctive alternate transcription of T-type calcium channel genes. This may result in easily induced oscillation of synchronization in the TC circuit, and they could be positively inclined to development of typical absence seizures. It is possible that some of the sporadic CAE patients may inherit both parents’ “strong” T-type calcium transcripts.

The mice of Cav3.1-deficient thalamus are resistant to the generation of SWD in response to GABA(B) receptor activation (34). On the other hand, transgenic murine lines overexpressing the cacna1g gene have pure absence seizures through genetic enhancement of the TC network (33). These findings suggest that “pure” absence seizure in human beings, such as typical CAE, may originate from enhanced T-current due to mutations or alternative transcripts of the three T-type calcium channel genes (Figure 1).

## Do T-Type Calcium Genes have a Dynamic Epigenetics Process Involved in the Mechanism of Absence Seizure?

Recent studies have identified epigenetic factors that may play a role in the pathogenesis of epilepsy (72, 73). DNA methylation is a crucial epigenetic modification of gene regulation and maintenance of genomic stability and it occurs primarily at cytosines, located 5′ to guanosine in a CpG dinucleotide. The interplay between genetic variants and epigenetics factors may be a widespread mechanism of transcriptional regulation. A mutation may have a significant impact on the local chromatin structure by modifying DNA methylation patterns or histone type recruitment (73). Therefore, understanding the underlying mechanism of the genetic origin of epilepsies requires us to identify the susceptibility alleles, and analyze gene–gene interactions, but also requires study of the interaction between genes and environment (74). The unique characteristics of typical absence seizures, which occur at a specific age and where subjects have no other identifiable neurologic abnormalities, may imply that genes and environmental factors combine to contribute to its distinctive pathological progression. Genetic variants are distributed across the genome in both coding and non-coding sequences. Selection pressure tends to decrease the frequency of genetic variants within exons, as compared to promoters and introns (75). However, the Cav3.2 gene seems to be unique in this regard. It has a high number of variants across the whole gene, with almost 1 SNP for every 30–48 base pairs in exons. These variants should have functional significance through effect on the gene expression as discussed above.

Methylation has been postulated as a mechanism for silencing tissue-specific genes in cell types where they should not be expressed. The Cav3.2 gene is tissue-specific; there are few or no transcripts of the Cav3.2 gene in white blood cells. However, adult T-cell leukemia patients have aberrant Cav3.2 gene mRNA transcripts, which may result from hypomethylation of Cav3.2’s CpG sites (76). More importantly, alpha 1H transcripts are 10–40 times more abundant in the human fetal brain when compared with an adult brain (69). Understanding the dynamic epigenetic process in different types of T-type calcium genes during development may be critical to understand the underlying mechanism of absence seizures, and may also explain why typical absence seizures remit in adults.

## Conclusion and Future Perspectives

The TC circuit sustains oscillatory burst firing. Due to the highly complex and widespread effects of different channel genes involved in this circuit, even small alterations in their expression or biophysical properties can induce pathophysiological changes in the brain with the potential to induce epileptic seizures (7).

T-type calcium channel genes are very important in the formation of SWD, and any modification of these types of genes will change the physiological characteristics of T-type channel subunits. All three of these subunits of T-type calcium genes have many alternative transcripts that when co-expressed in the same neuron, may enable some of them to enhance the TC synchronous oscillatory burst-firing circuit, and potentially initiate absence seizures. Certain variants may be expressed in an age-specific pattern and may help explain the well-known age dependency of typical absence epilepsy. Modification by epigenetic processes such as DNA methylation may also play an important pathogenic role and may help explain the very complex genetics of this disorder, which rarely follows simple Mendelian patterns. Future research exploring the mechanism of absence seizures should not focus exclusively on the gain or loss of function from missense mutations, but should also consider genetic profile complexity. Gain or loss of function mutations, and other variations that alter both quantity and quality of transcripts, and methylation status of isoforms of T-type calcium channel proteins might be of equal importance in understanding the pathological mechanism of absence seizures.

## Conflict of Interest Statement

The authors declare that the research was conducted in the absence of any commercial or financial relationships that could be construed as a potential conflict of interest.
